# DAPL1 is activated by Np63 and GRα and regulates lipid metabolism

**DOI:** 10.1007/s00109-025-02636-8

**Published:** 2026-01-31

**Authors:** Heung-Seok Bae, Yong-In Kim, DongWook Kim, Ki-Hoan Nam, KyungSook Han, SunJo Kim, SungWon Kwon, Je-Yoel Cho

**Affiliations:** 1 Department of Biochemistry, College of Veterinary Medicine, BK21 FOUR Future Veterinary Medicine Leading Education and Research Center, Research Institute for Veterinary Science, and, Seoul National University , 1 Gwanak-Ro, Gwanak-Gu, Seoul, 08826 Republic of Korea; 2 Comparative Medicine Disease Research Center (CDRC), Science Research Center (SRC), Seoul National University, Seoul, 08826 Republic of Korea; 3 Laboratory Animal Resource & Research Center (LARRC), KRIBB, Yeongudanji-Ro 30, Cheongju, Chungbuk 28116 South Korea; 4 Department of Computer Engineering, Inha University, Incheon, 22212 South Korea; 5 College of Pharmacy, Chonnam National University, Gwangju, 61186 South Korea; 6 College of Pharmacy, Seoul National University, Seoul, 08826 South Korea; 7 Cambridge Centre for Proteomics, Department of Biochemistry, University of Cambridge, Cambridge, CB2 1QR UK

**Keywords:** DAPL1, Np63, GRα monomer, Fdft1, Pcyt1a, Sptlc1

## Abstract

**Abstract:**

Non-small cell lung cancer (NSCLC) accounts for about 80% of all lung cancer occurrences and is divided into largely Adenocarcinoma (LUAD), Squamous cell carcinoma (LUSC), and Large cell carcinoma. In this study, using RNA-seq data between cancer tissues and adjacent normal tissues of 5 LUAD and 4 LUSC patients, we found that a Death associated protein like 1 (DAPL1) was highly expressed in squamous cell carcinoma but not in adenocarcinoma. Through the RNA-seq analysis, we found that lipid metabolic pathway genes Fdft1, Pcyt1a, and Sptlc1 correlate well with DAPL1 level changes in LUSC. We also found that Dapl1 was activated by Np63 and GRα monomer transcription factors. We generated a DAPL1 knockout mouse, which shows body weight and hair color changes, implying the role of DAPL1 in lipid metabolism. Our data show that Np63, GRα transcription factors activate DAPL1, and it is predicted to contribute to cellular acidification by regulating lipid metabolism based on mRNA-seq data and DAPL1 KO mice.

**Key messages:**

- This study uses RNA-seq data between cancer tissues and adjacent normal tissues of 5LUAD and 4LUSC patients.
- We found that a Death associated protein like 1 (DAPL1) was highly expressed in squamous cell carcinoma but not in adenocarcinoma.
- We also found that Dapl1 was activated by Np63 and GRα monomer transcription factors.
- The RNA-seq analysis found that lipid metabolic pathway genes Fdft1, Pcyt1a, and Sptlc1 correlate well with DAPL1 level changes in LUSC.
- We generated a DAPL1 knockout mice. DAPL1 KO mice were bred at two institutions (KRIBB for phonotypes, and SNU for functions). Interestingly, in KRIBB, DAPL1 KO body weight was lower than WT, and in SNU, DAPL1 KO body weight was higher than WT. In the investigation of mouse chow from the two institutions, differences in choline were noted. When chow with differences only in choline was produced and applied to new DAPL1 KO mice, the same results were obtained.
- Through RNA-seq data and body weight changes in DAPL1 KO mice, we report that DAPL1 regulates cholesterol, PC, and SM through changes in mRNA of Fdft1, Pcyt1a, and Sptlc1.

Our data show that Np63, GRα monomer transcription factors activate DAPL1, and it is predicted to regulate lipid metabolism based on mRNA-seq data and DAPL1 KO mice.

**Supplementary information:**

The online version contains supplementary material available at 10.1007/s00109-025-02636-8.

## Introduction

Lung cancer is ranked highest cancer occurrence (2 480 675 (12.4%)), leading to the highest mortality worldwide in 2022 (1 817 469 (18.7%)). Lung cancer is divided into small cell lung cancer (SCLC, 15%) and Non-small cell lung cancer (NSCLC, 80%: Adenocarcinoma (LUAD) 40%, Squamous cell carcinoma (LUSC) 25%, Large cell carcinoma 15%) and others (5%) [1–3]. While analyzing mRNA-seq data between cancer tissues and adjacent normal tissues from 4 LUSC and 5 LUAD patients, DAPL1, which was not expressed in normal tissues and LUAD, was found to be highly expressed in 2 LUSCs, moderately expressed in 1 patient, and not expressed in 1 patient. By leveraging variation in DAPL1 expression, we identified transcripts whose expression patterns correlated with DAPL1 in the mRNA-seq dataset, thereby enabling inference of *DAPL1*’s functional roles. DAPL1, also known as Early Epithelial Differentiation Associated (EEDA), was discovered by Sun et al. They reported that it is expressed in early differentiating cells of stratified squamous epithelia associated with keratinocyte differentiation, such as in the corneal epithelium, epidermis, tongue epithelium, growing hair follicle, and nail matrix [4]. Since its discovery, the role of DAPL1 has been reported in several tissues, with extensive research being conducted in the eye. DAPL1 SNPs were related to a female-specific Age-related Macular Degeneration (AMD) susceptibility locus [5]. DAPL1 was identified as a candidate gene related to posterior subcapsular congenital cataracts [6]. It is also reported that DAPL1 has an anti-proliferative effect in the retinal pigment epithelium (RPE) cell and is regulated by (-) mitf-MSI2-pre-miR7 [7] [8]. Dapl1 loss inhibits RPE autophagy and produces age-related retinal pathology [9]. DAPL1 is reduced in proliferative vitreoretinopathy (PVR), and DAPL1 deficiency promotes Epithelial–mesenchymal transition (EMT) in RPE cells in mice [10]. Serum testosterone level was elevated in the DAPL1 null mice [11]. In the immune system, Dapl1 was predominantly expressed in CD8^+^ T cells and involved in the negative regulation of CD8^+^ T cell expansion in responses to chronic infection and cancer [12]. When searching the substantia nigra and Parkinson’s disease data sets in the Gene Expression Omnibus (GEO) database, DAPL1 has been shown to play a key role in the occurrence and development of Parkinson’s disease [13]. Regarding cancer, in comparing gene expression patterns between Birt-Hogg Dubé syndrome (BHDS) derived renal tumors and other renal tumors, Dapl1 was expressed at a high level in BHDS-derived renal tumors [14]. Hepatoblastoma, a rare embryonal tumor of the liver, reflects whole-chromosome aneuploidies, especially the 2q24 region (including the DAPL1 gene). DAPL1 is one of the significantly upregulated 5 genes in hepatoblastoma [15]. In melanoma, it showed a cancer suppressing function by increasing P21 stability [16]. Taken together, the evidences indicate that DAPL1 functions as an anti-proliferative regulator during early differentiation in ocular and melanoma, but may acquire oncogenic properties in the context of tumorigenesis.

In normal human tissues, DAPL1 is expressed in areas where keratinization occurs (hair, nails, skin, cornea, tongue, vagina, esophagus, etc.) [4], areas where melanin synthesis occurs (skin, hair, etc. with melanocytes, and retinal pigment epithelium (RPE)) [16] [8], and areas where catecholamine (dopamine, epinephrine, norepinephrine) synthesis occurs (kidney [17, 18], adrenal medulla [19, 20], midbrain (substantia nigra) [21, 22], hypothalamus [23, 24], testis Leydig cell [25]), and expression is particularly high in the eyes [11]. We discovered one commonality from the expression sites of DAPL1. The expression of enzymes such as melanin synthesis enzyme (tyrosinase, optimum pH 6.5, [26]), dopamine synthesis enzyme (tyrosine hydroxylase, optimum pH 6.9, [27]), keratinization enzymes (retroviral-like aspartic protease 1 (ASPRV1, pH 5.5–6.5 [28]), b-glucocerebrosidase (GBA1, pH 5–6 [29]), neutral ceramidases (nCDases, pH 6.0–7.5 [30]), bleomycin hydrolase & other neutral proteases pH ~ 6.0 [31]) requires an acidic environment of pH 6–7 or pH 5–6. In particular, the eye is an organ that requires oxygen deprivation because it has only a nucleus and no organelles (the lens of the eye does not even have a nucleus) to increase light transmittance and it supplies energy through glycolysis [32–34]. Our experimental results showed that DAPL1 changes the composition of cell membrane lipids, thereby reducing oxygen permeability, thereby maintaining the cytoplasm as slightly acidic.

**Scheme 1:**
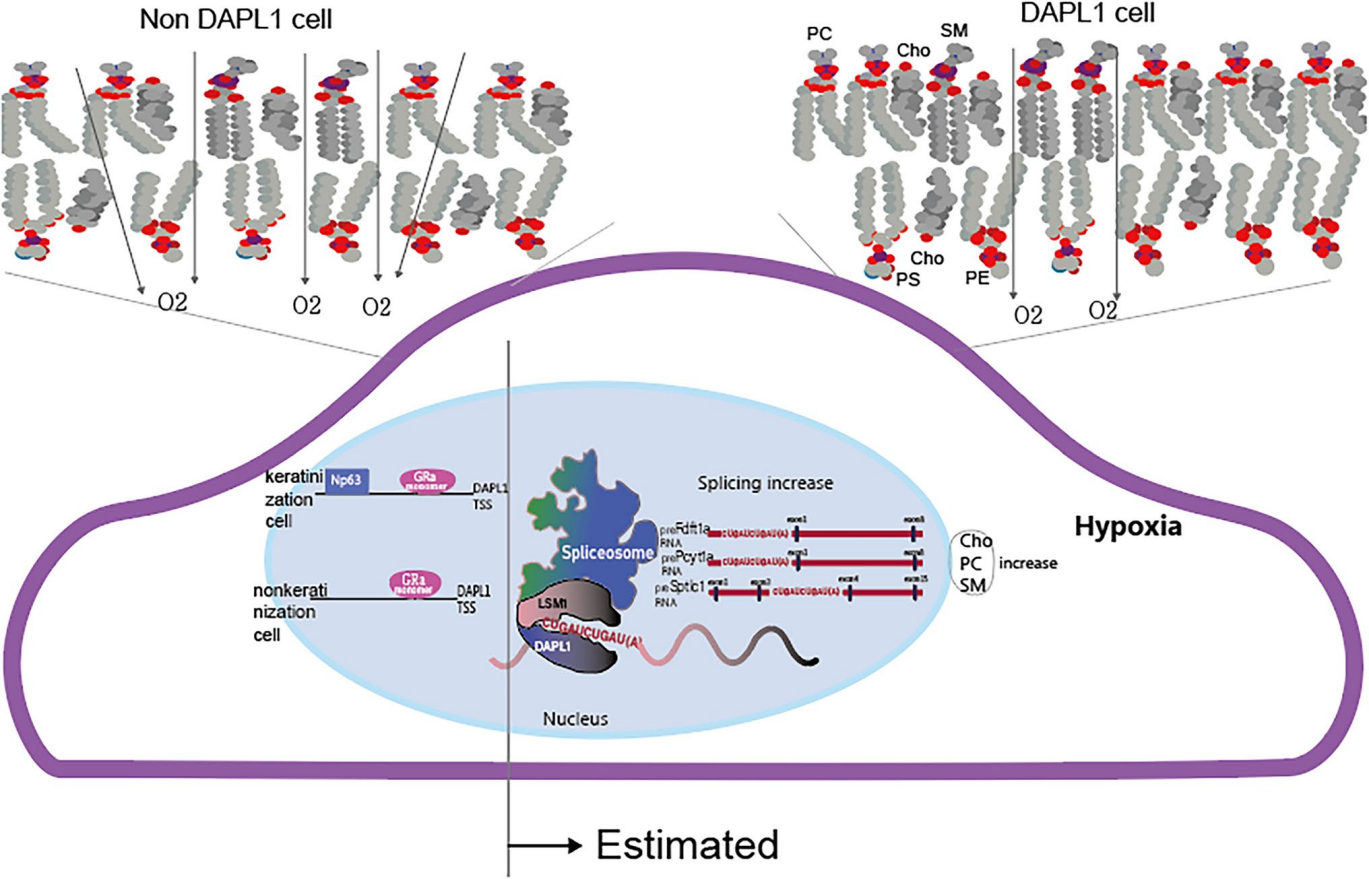
Schematic diagram

In this study, using our RNA-seq data between cancer tissues and adjacent normal tissues, we found that Dapl1 was highly expressed in LUSC but not in LUAD. Based on NCBI GEO Profiles, the transcription factors of DAPL1 were confirmed to be Np63 and GRα monomer. We also found that Fdft1, Pcyt1a, and Sptlc1 expressions were correlated with Dapl1. We further analyzed the regulation and the role of DAPL1 in DAPL1 KO mice. DAPL1 KO mice showed changes in body weight and hair color depending on the difference in choline components, indicating that the function of DAPL1 is related to the function of choline components. Based on the literature on metabolomics and lipidomics research methods in lung cancer [35, 36], and literature analyzing metabolites and lipids in NSCLC patients [37, 38], we used LC/MS to measure lipid changes in mouse eyes with high DAPL1 expression**.** Also, the schematic diagram summarizing the results of our experiment is as follows (Scheme 1).

## Schematic explanation

Since the master transcription factor of keratinization is Np63, DAPL1 is activated by Np63 and GRα monomer in keratinized cells, and by GRα monomer in nonkeratinized cells. DAPL1 forms a dimer with LSM1, binds to the CAGAUCAGAU array of pre-Fdft1 RNA, pre-Pcyt1a RNA, and pre-Sptlc1 RNA, increases splicing by the spliceosome, and increases the expression of mFdft1, mPcyt1a, and mSptlc1. These play a role in controlling the rate of synthesis of Cho, PC, and SM, resulting in increased expression of Cho, PC, and SM. PC and SM are mainly responsible for the outer membrane of the cell double membrane, and when Cho binds to PC, the permeability of oxygen and water decreases, which leads to acidification of the inside of the cell.

## Materials and methods

### Cell culture

HCC95 and SKMES1 LUSC cells were purchased from Korean Cell Line Bank (KCLB, cellbank.snu.ac.kr, Seoul Korea). The cells were cultured in RPMI1640 (HyClone Cat.no SH30027.01, Logan, UT, USA) supplemented with 10%FBS (AbFRONTIER Cat.no A19001, Seoul Korea) in a 5% CO_2_ humidified incubator at 37℃. For transfection, HCC95, SKMES1 cells are seeded 3*10^5^/well in 6 well plates, 24 h later, DAPL1siRNA or pcDNA3.1-DAPL1-DYK vector transfected. After incubation at 37℃ for 24 h or 48 h, 72 h, cells are used for experiments. (PCR, RT-PCR, Luciferase assay, Colony formation assay). For the hypoxia experiment, an incubator with 94% N_2_, 5% CO_2_, 1% O_2_ was used. (Whitley H35 hypoxystation, don whitley scientific).

### Plasmid, siRNA, and transfection

A pcDNA3.1-DAPL1 ORF DYK tag vector (NM_001017920.2) was purchased from ORIGEGE (Rockville, USA). DAPL1siRNA and Np63siRNA were customized from BIONEER (Daejeon, Korea) DAPL1siRNA Sense: AAACAAGUGCCAUUGCAAAUGUU Antisense: AACAUUUGC AAUGGCACUUGUUU. Np63siRNA Sense: AACCAUGAGCUGAGCCGUGAAUU Antisense: AAUUCACGGCUCAGCUCAUGGUU. Transfection for plasmid, siRNA is performed using Lipofectamine 3000, Lipofectamine RNAiMAX reagent (Invitrogen, Massachusetts, USA).

#### RT-PCR, PCR

The RT-PCR was performed as previously reported [39]. Briefly, total RNA was extracted from the HCC95, SKMES1 cells, and mouse tissues using the TRizol reagent (Invitrogen, 15,596,018). Target mRNA was amplified with SYBR reagent (cat. no. 1725124, Bio-Rad) and Go-taq polymerase (M829B, PROMEGA) using RT–PCR machine (CFX Connect Real-Time System, Bio-Rad) and for PCR (T100 Thermal Cycler, Bio-Rad). The primers used for the RT-PCR, PCR was listed in the Supplement data: Table 6.

### Luciferase assay

DNA of HEK293T cells is extracted by DNeasy (QIAGEN, Blood & Tissue Kit (250) Cat. No 69506). DAPL1 promoter 1 kb is subjected to PCR using Forward primer: ATA–Sac1-DAPL1 promoter (29 bp) and Reverse primer: ATA-Nhe1-DAPL1 promoter (29 bp). PCR products are gel extracted (QIAquick Gel extraction Kit (50), 28,704, QIAGEN) The gel extraction product is subjected to TA cloning. (pGEM-T Vector, A362A PROMEGA) DH5α transduction and white colony selection, miniprep, then extract plasmid (DNA-spin, Plasmid DNA Purification Kit, 17,098, iNtRON). Sac1, Nhe1 Restriction enzyme cutting on TA vector including target and pBV-Luc vector (Addgene), gel extraction, DNA concentration measure, ligation (T4 DNA Ligase, M180A, Promega), DH5α transduction, Ampicillin resistance colony miniprep, sequencing (BIONICS, Seoul Korea), midiprep (Xtra Midi EF, LOT 2207–005, MACHEREY–NAGEL, Germany). By measuring DNA concentration, plasmid preparation for HEK293T cell transfection is made. For luciferase assay, EZ Luciferase Assay system kit (EZ040S, Enzynomics) and 1420 Multilabel Counter (VICTOR3, PerkinElmer) are used.

### Colony formation assay

Colony formation assays were done as previously reported [40]. Briefly, 1,000 cells (HCC95), 1,000 cells (SKMES1) per well were plated in 6 well plates and after 6 ~ 7 days, cells were treated with control siRNA 20,10 pmol/well and DAPL1 siRNA 20,10 pmol/well.

### DAPL1 KO mouse and animal experiments

DAPL1 KO mice were generated using the Crisper-Cas9 system with the sgRNA sequence 5′ TGTTTCCAGTGAAAGCTGGAGGG 3′, 3’ ATGAACCAGGAACAAACCAAAGG 5’ on C57BL/6N-Tac background mice by the Korea Research Institute of Bioscience and Biotechnology (KRIBB). DAPL1 KO mice were created by deleting a 127 bp sequence of exon 2 and intron of the DAPL1 gene. Knockout of the DAPL1 gene was confirmed with forward primer TCCCTGCTCTGGCTTCCTTA, reverse primer CCAACAGGAGTGTTCCAGG G (406 bp). All animal care and experiments were performed in accordance with the permits and standards of SNU IACUC. (approval #SNU-181114–2-2, SNU-210323–4-3, SNU-221018–1-1).

### Lipidomics

Eye sample extraction was performed following Matyash’s methyl *tert*-butyl ether (MTBE) extraction method [41]. The samples were washed with PBS and snap-frozen in liquid nitrogen immediately after harvesting. Each eyeball was mixed with 350 μL of ice-cold water and homogenized using a bead beater (Precellys Evolution, Bertin Technologies, Rockville, MD, USA) at 7,200 rpm for 2 × 30 s under dry ice–precooled conditions. Approximately 50 μL of homogenate from each sample was pooled to prepare a QC sample, which underwent the same extraction procedure. A 250 μL aliquot of homogenate from each sample was transferred to a new 2 mL tube, followed by the addition of 300 μL of − 20 °C methanol (Supelco, LC–MS grade) and 1,000 μL of − 20 °C MTBE (Sigma-Aldrich, HPLC grade). Biphasic extraction was performed on a 4 °C ThermoShaker for 1 h at 1,500 rpm. After centrifugation at 16,000 g for 10 min at 4 °C, 900 μL of the upper lipophilic layer was collected and dried under a gentle stream of nitrogen. The dried extract was reconstituted in 200 μL of methanol: toluene (9:1, v/v) and injected into the LC–MS system.

Two microliters of each sample were injected into an Agilent 1290 ultra-performance liquid chromatography (UPLC) coupled to an Agilent 6530 hybrid quadrupole Time of Flight mass spectrometer, equipped with an Acquity UPLC CSH C18 column (130 Å; 1.7 μm; 2.1 × 100 mm). The column temperature was maintained at 65 °C, and the flow rate was set to 0.6 mL/min. Mobile phase A consisted of acetonitrile: water (6/4, v/v), and mobile phase B consisted of 2-propanol: acetonitrile (9/1, v/v), both containing 10 mM ammonium formate and 0.1% formic acid. Chromatographic separation was performed using the following gradient of mobile phase B, with a 4-min post-run: 0 min, 15%; 2 min, 30%; 2.5 min, 48%; 11 min, 82%; 11.5 min, 99%; 12 min, 99%; 13 min, 15%; and 16 min, 15%. Data-dependent acquisitions were used for lipid identification and quantification on both positive and negative ionization fields. Data processing and lipid identification were performed using MS-DIAL ver. 4.9. Lipids were annotated at the bond-type level in accordance with the Lipid Standards Initiative guidelines [42], based on their m/z accuracy (accurate mass score > 900) and MS/MS spectral similarity (reverse dot-product score > 700). When duplicate annotations were generated from different ionization modes, the feature with the higher overall identification score was retained for subsequent analysis. Detailed analytical mass spectrometry parameters and data-processing method are provided in Supplement Tables 1 and 2, which followed a previous research [43].

### Statistics

The t-test and one-way ANOVA test were implemented to compare gene expression between groups. (GraphPad Prism 9.0) The number of asterisks between the two groups indicates the degree of statistical significance. Multivariate analysis was conducted using MetaboAnalyst 6.0. Prior to analysis, the pre-processed lipidomic data were median-normalized, log-transformed, and Pareto-scaled. Lipid features with more than 50% missing values were removed, and the remaining missing values were imputed with one-fifth of the minimum positive value for each variable.

## Results

### Dapl1 mRNA is highly expressed in LUSC

**Fig. 1:**
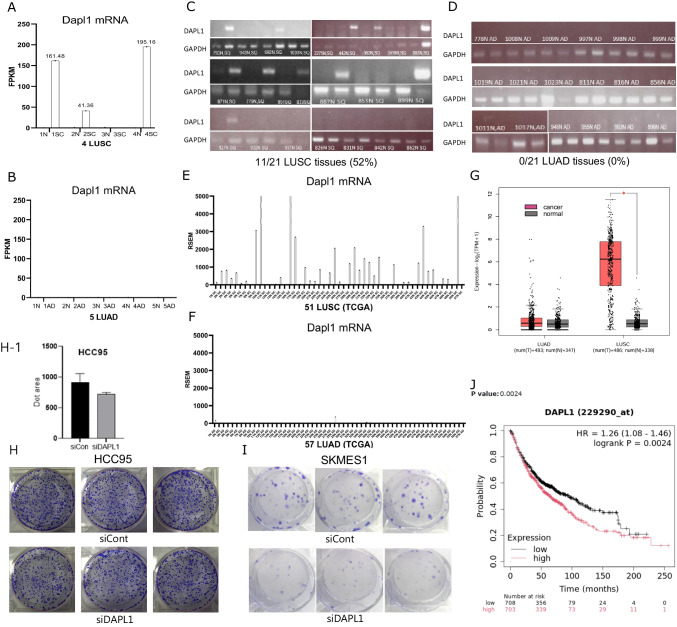
Dapl1 mRNA in LUSC is highly expressed (**A**) Three out of our four LUSC patients were upregulated in Dapl1 expression compared with adjacent normal (FPKM = fragments per kilobase per million reads). (**B**) Five LUAD have not detected or very low levels of Dapl1 expression in RNA-seq data. (**C**) Twenty-one Human LUSC and (**D**) twenty-one Human LUAD tissues were analyzed to determine Dapl1 expression level by PCR. Dapl1 was upregulated in 11 LUSC (52%), while no expression was observed in LUAD. (**E**) Expression levels of Dapl1 in TCGA (Cancer tissue and nearby normal tissue from the same patient) database, Dapl1 were upregulated in 36 among 51 LUSC (70.6%, mean RSEM 1142), but (**F**) Dapl1 was upregulated in 3 among 57 LUAD (5%, mean RSEM 13), RSEM (RNA-Seq by Expectation–Maximization) reports transcripts per million mapped reads (TPM). (**G**) Graph comparing Dapl1 expression in normal tissue and LUSC, and normal tissue and LUAD from GEPIA2 (GEPIA 2). (**H**) Colony formation assays were done by seeding HCC95 cells (1,000 cells/well) (**H-1**) Graphing HCC95 colony formation assay results using Clono-counter, and (**I**) SKMES1 cells (1,000 cells/well) on 6 well plates. Dapl1 knockdown by siRNA dramatically reduced the colony formation. (**J**) Kaplan–Meier survival curve shows that the higher the Dapl1 expression, the worse the prognosis of lung cancer. Data was drawn from https://kmplot.com

In our analysis of RNA sequencing data from cancerous and adjacent normal tissues of five patients with lung adenocarcinoma (LUAD) and four with lung squamous cell carcinoma (LUSC), Dapl1 expression was observed exclusively in squamous carcinoma. It was absent in adenocarcinoma and normal tissues. Within the squamous carcinoma cohort, Dapl1 exhibited high expression levels in two of the four LUSC patients (FPKM > 150), moderate expression in one (FPKM > 25), and was undetectable in another (Fig. 1A, B). Further investigation using PCR analysis of Dapl1 mRNA across 21 patients with LUSC and LUAD respectively, revealed its presence in the tumor tissues of 11 LUSC patients (52%) but not in any LUAD patients (Fig. 1C, D). Analysis of the TCGA dataset (version dated 2016_01_28 from firebrowse.org) indicated that 36 out of 51 LUSC patients (70.6%) exhibited higher Dapl1 expression compared to their corresponding normal tissues, with a mean RSEM value of 1,142 (Fig. 1E). In contrast, only 3 out of 57 LUAD patients showed elevated Dapl1 levels above those in normal tissue (5.26%, mean RSEM value of 13) (Fig. 1F).These findings were corroborated by data from the GEPIA2 database (GEPIA 2), confirming higher Dapl1 transcript levels in LUSC but not in LUAD (Fig. 1G). Consequently, our study confirms the pronounced expression of Dapl1 in LUSC. Subsequent experiments focused on the role of DAPL1 in LUSC revealed that its knockdown via siRNA diminished colony formation in HCC95 and SKMES1 LUSC cell lines (Fig. 1H, H-1, I). These observations suggest that DAPL1 facilitates the proliferation of LUSC cells, supporting evidence from survival analysis via the Kaplan–Meier plotter (kmplot.com) which shows a decrease in survival as DAPL1 levels increase (Fig. 1J).

### DAPL1 is regulated by Np63

**Fig. 2:**
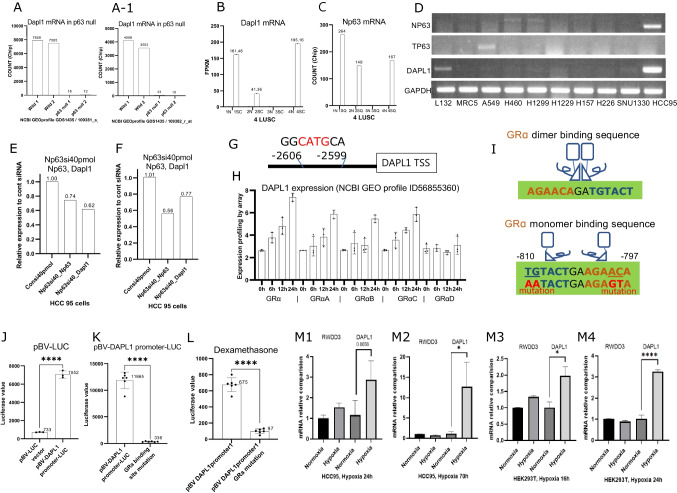
Np63, GRα monomer are the transcription factors of DAPL1 and Dapl1 is increased in hypoxia depending on the situation. (**A**) When transcription factor Np63 was knocked out in MG-U74B skin cells, Dapl1 disappeared. (GDS1435/109381,109382, GEO Profiles, NCBI). (**B**)(**C**) Dapl1, Np63 expression in RNA-seq data of our 4 LUSC patients (cancer tissue and around normal tissue). (**D**) Among lung cancer cell lines, Np63, a transcription factor, was confirmed to be expressed in HCC95, which expresses Dapl1. (L132: Human cervix carcinoma. Originally derived from a human embryonic lung). (**E**)(**F**) When Transfecting the HCC95 cell with Np63siRNA, the expression of Np63, Dapl1 mRNA is identified by RT-PCR. (**G**) Estimated Np63 binding site in the DAPL1 promoter region based on a recent paper [44]. (**H**) When osteosarcoma cells were transfected with GRα, GRα A, B, C, and D, Dapl1 increased in GRα, GRα A, B, and C over time, but did not increase in D (ID 56855360, GEO Profiles, NCBI). (**I**) The typical GRα dimer binding sequence and the monomer binding site at positions −797 to −810 bp of the DAPL1 promoter, for mutation experiments, mutate TGAA, ACGT. (**J**) Luciferase assay results, empty vector vs DAPL1 promoter vector, (**K**) DAPL1 promoter vector vs mutant vector, (**L**) DAPL1 promoter vector vs mutant vector-Dexamethasone 1uM, 3uM addition. (**M1,2**) HCC95 and (**M3**,**4**) HEK293T cells plated in 6 wells were grown under hypoxia conditions (N_2_ 94%, CO_2_ 5%, O_2_ 1%) incubator (Whitley H35 hypoxystation), after 18 h, 24 h, 70 h, it is compared with the control group grown under normal conditions by RT-PCR

To find the regulatory mechanism of Dapl1 activation in LUSC, we searched transcription factors cis-acting on Dapl1 enhancers. When Np63 was knocked out in MG-U74B skin cells (NCBI, GEO Profiles, ID 14185288, 14185289), DAPL1 expression almost disappeared (Fig. 2A, A-1). In our RNA-seq data of 4 LUSC patients, the expression pattern of Np63 correlated well with Dapl1 expression (Fig. 2B, C). When the expression of Dapl1 was investigated in lung cancer cell lines, Dapl1 was expressed in HCC95 LUSC cells in which Np63 was also expressed (Fig. 2D). When Np63 was knocked down by siRNA in HCC95 cells, Dapl1 expression was downregulated (Fig. 2E and F). Based on a recent report revealing Np63 binding sequences [44], we were able to find a Np63 binding site in an upstream enhancer region of −2,606 to −2,599 to the transcription start site of Dapl1 (Fig. 2G). In a paper introducing the IL13-Np63 axis, SERPINB1 & 4, and DAPL1 are reported as genes that increase when the esophageal squamous epithelium is stimulated with IL13 and decrease when Np63 is knock down [45]. According to a report by NCBI Gene, P63 has 13 isomers and is divided into TAp63 and deltaNp63, of which deltaNp63 is divided into three subisomers, alpha, beta, gamma [46]. In ChEA Transcription Factor Targets 2022, which was created based on data from ChIP-seq and transcription factor binding studies, on the page (Gene Set—P63-20,808,887-KERATINOCYTES-HUMAN), among 1596 P63 target genes, DAPL1 is included. All of the above results indicate that the Np63 transcription factor regulates the Dapl1 expression.

### DAPL1 is regulated by GRα monomer transcription factors and in hypoxia

Another transcription factor on the Dapl1 regulatory region was found in NCBI GEO Profiles ID 56855360. When glucocorticoid receptor (GR) isomers were expressed in osteosarcoma cells, the expression of Dapl1 was increased by GRα, GRαA, B, and C, but not in GRαD (Fig. 2H). Normally, GRα functions as a dimer. The Dapl1 promoter region appears to have two binding sites for two GRα monomers. These are not dimer binding sites for GRα.

We aimed to confirm this by mutating the GRα binding site on the promoter of Dapl1 and performing a luciferase assay. We identified the GRα monomer binding site in −810 to −797 to transcription start site (TSS) of Dapl1 and mutated them as shown in Fig. 2I. After transfecting vectors of GRα binding sites mutated and wild-type into HEK293T, promoter luciferase assays were done. Initial transfection of the Dapl1 promoter vector showed robust activity compared to the basic vector indicating GRα monomer binding site exists on the Dapl1 promoter cloned (Fig. 2J). The Dapl1 promoter luciferase vector with GRα monomer site mutated resulted in a dramatic decrease of the luciferase activity (Fig. 2K). The mutated form of the luciferase vector did not respond to the dexamethasone treatment, indicating that the GRα monomer binding sites on the Dapl1 promoter are important for responding to glucocorticoid and GRα signaling (Fig. 2L).

In order for the GRα to become a monomer naturally, sumoylation must occur at the Lysine (K) 703 position of the ligand binding domain (LBD) of GRα [47]. In addition, RWD-Containing sumoylation enhancer (RSUME(= RWDD3)) is known to increase in cell crisis conditions such as hypoxia, virus invasion, etc. [48]. Therefore, we tested whether Dapl1 expression in the cells changes in response to hypoxia. Dapl1 expression was significantly increased when the HCC95 cells and HEK293T cells were under hypoxia conditions (Fig. 2M1, 2, 3, 4).

### DAPL1 regulates lipid synthesis enzymes.

**Fig. 3:**
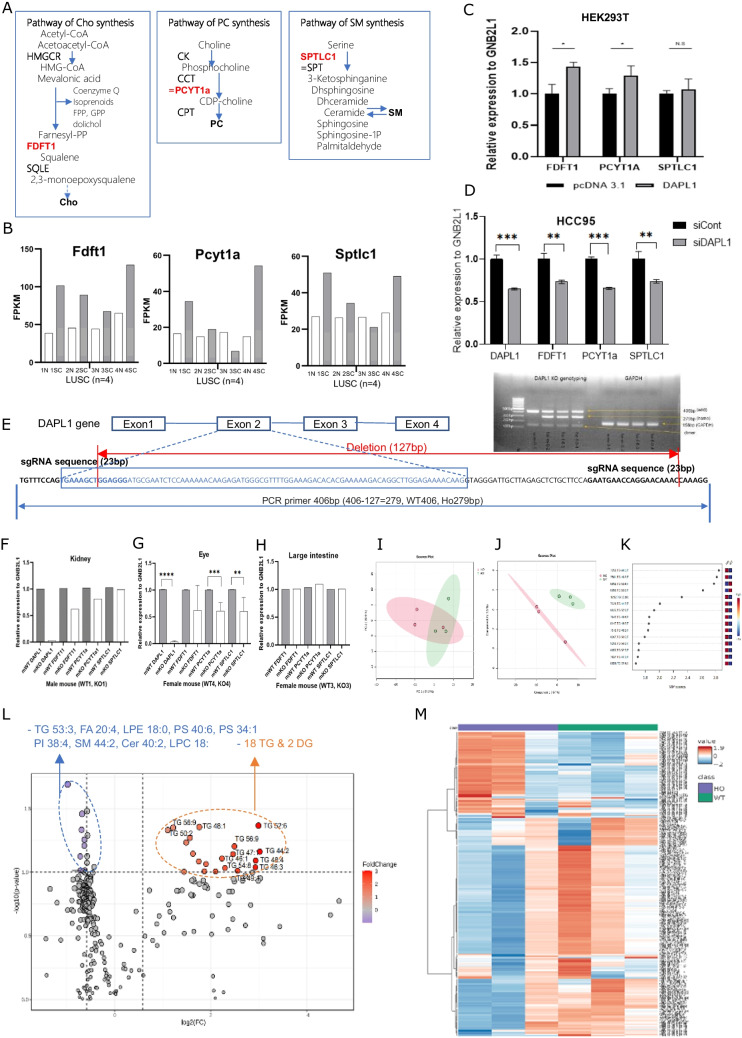
DAPL1 changes lipid synthesis enzymes. (**A**) Lipid synthesis pathways related to Fdft1, Pcyt1a, Sptlc1 genes. (**B**) Among the 10 genes, expression comparison of the finally selected Fdft1, Pcyt1a, and Sptlc1 genes in normal and adjacent cancer tissues of four LUSC patients. (**C**) When Dapl1 was overexpressed in HEK293T cells, changes in Fdft1, Pcyt1a, and Sptlc1 mRNA were confirmed by RT-PCR. (**D**) When Dapl1 was knockdown in HCC95 cells, changes in Dapl1, Fdft1, Pcyt1a, and Sptlc1 mRNA were confirmed by RT-PCR. (**E**) Schematic diagram of DAPL1 knockout using CRISPER-Cas9 system. 127 bp is deleted between exon2 and intron. The distance between two primers is 406 bp. Figure of genotyping results of generated mice. (**F**) In the kidney tissue of the DAPL1 KO male mouse, Dapl1, Fdft1, Pcyt1a, and Sptlc1 mRNA were confirmed by RT-PCR. (**G**) In 4 WT, 4 DAPL1 KO female mice’s eyes (high DAPL1 expression), Dapl1, Fdft1, Pcyt1a, and Sptlc1 mRNA were confirmed by RT-PCR. (**H**) In 3 WT, 3 DAPL1 KO female mice’s large intestines (no DAPL1 expression), Fdft1, Pcyt1a, and Sptlc1 mRNA were confirmed by RT-PCR. (**I**) After LC/MS analysis of 297 types of lipids in the eyes of male mice 3 WT and 3 DAPL1 KO, the principal component analysis (PCA) plot confirms the division into two groups. (**J**) In Partial least squares-discriminant analysis (PLS-DA), a supervised learning model, it was confirmed that the two groups were clearly divided. (**K**) From the importance scores, lipids of the TG class appeared to act as a major factor in model training. (**L**) In the Volcano plot, 18 types of TG & 2 types of DG have up-regulated (tendency shown in lipid class), TG 53:3, FA 20:4, LPE 18:0, PS 40:6, PS 34:1, PI 38:4, SM 44:2, Cer 40:2, LPC 18:0 were down-regulated (No tendency in lipid class) fold change > 1.5, -log(p) > 1.0. (**M**) In Heatmap analysis, an overall difference between the blue (Ho) and green (WT) groups could be confirmed

In our mRNA-seq dataset, which includes samples from five LUAD and four LUSC patients, Dapl1 expression was absent in the normal tissues associated with both LUAD and LUSC. Among the LUSC patients, two exhibited significant upregulation of Dapl1, one showed mild upregulation, and one displayed no change in expression levels (Fig. 1A). Prompted by this expression pattern, we conducted a search for genes with similar expression profiles. Ten genes were identified as exhibiting similar expression patterns (see Supplemental Data: Table 3). Of these, three genes—Fdft1, Pcyt1a, and Sptlc1—were selected for further analysis. The products of these genes are enzymes that play crucial roles in the biosynthesis of cholesterol (Cho), phosphatidylcholine (PC), and sphingomyelin (SM), respectively (Fig. 3A, B). To see whether these 3 genes respond to Dapl1 expression, we transfected DAPLl1 into HEK293T cells and measured the 3 transcripts of the genes using RT-PCR. Fdft1 and Pcyt1a expression was significantly increased, although Sptlc1 tends to increase but did not show statistical significance (Fig. 3C). When Dapl1 was knocked down in HCC95 cells using Dapl1 siRNA, those 3 genes were significantly decreased (Fig. 3D).

To test whether Dapl1 Knock-out changes the expression of these 3 lipid enzyme genes in vivo, DAPL1 KO mice were produced using the CRISPR-Cas9 system (Fig. 3E). DAPL1 KO mice and wild born through hetero-hetero mating were used in these experiments, genotyping was done using primers of wild-type 406 bp, homo 279 bp (deletion length 127 bp). Dapl1 KO mice showed a decrease of Fdft1 transcript in kidneys (Fig. 3F) and Pcyt1a and Sptlc1 transcripts in eye tissues (Fig. 3G), where the Dapl1 expression is high in wild type. There were no differences in these 3 genes in the large intestines, which do not express Dapl1, between the WT and KO mice (Fig. 3H).

To assess lipid alterations, lipid profiles were quantified in the eyes of three male wild-type and three DAPL1 knockout (KO) mice using liquid chromatography-mass spectrometry (LC/MS). A total of 297 lipid species were annotated, of which 236 were detected in positive ion mode and 61 in negative ion mode. Lipids detected in positive ion mode primarily included carnitines, (hexosyl)-ceramides, sphingomyelins (SMs), phosphatidylcholines (PCs), diglycerides (DGs), and triglycerides (TGs). Free fatty acids and additional phospholipid classes were mainly annotated in negative ion mode. The principal component analysis (PCA) plot, an unsupervised machine learning technique, illustrated a separation trend between the two groups (Fig. 3I). Furthermore, in the partial least squares-discriminant analysis (PLS-DA), a supervised learning approach, the groups were distinctly segregated (Fig. 3J). Notably, TGs were identified as a critical factor in the model training (Fig. 3K). In the analysis of a volcano plot with a fold change threshold of 1.5 and a -log(p-value) greater than 1.0, 18 types of TG and 2 types of DGs exhibited increased levels in DAPL1 knockout (KO) mice compared to wild-type (WT) controls. Conversely, levels of 9 lipid types were reduced. Notably, the decreased lipids did not display consistent trends across any specific lipid classes (Fig. 3L). In the heatmap analysis, there was a clear distinction between the KO (blue) and WT (green) groups. Characteristically, the samples from the KO group (3) showed a closer alignment with the trends observed in the WT group (Fig. 3M). The absence of discernible trends among the decreased lipid classes may be attributed to significant sample variation, the small sample size, or delays akin to those experienced in RNA experiments. Notably, a reduction of PCYT1A, the principal enzyme for phosphatidylcholine (PC) synthesis, by 50% in Caco2 cells resulted in an increase in TG [49]. Similarly, experiments in CHO MT58 cells demonstrated that a decrease in PC levels related with increases of 34% and two fold in the activities of TG-synthesizing enzymes DGAT and GPAT, respectively [50]. Furthermore, in mice exposed to perfluorooctanoic acid, elevated TG levels were observed alongside reductions in PC, SM, and cholesterol esters [51]. Conversely, analyses of 25 colon cancer patients revealed decreased TG and increased PC, SM, and cholesterol in cancerous tissues compared to normal counterparts [52]. These findings suggest that the upregulation of TG in DAPL1 KO mice may be driven by reductions in PC, SM, and cholesterol.

### Dapl1 Knock-out mouse showed changes in body weight and hair according to changes in choline amount

**Fig. 4:**
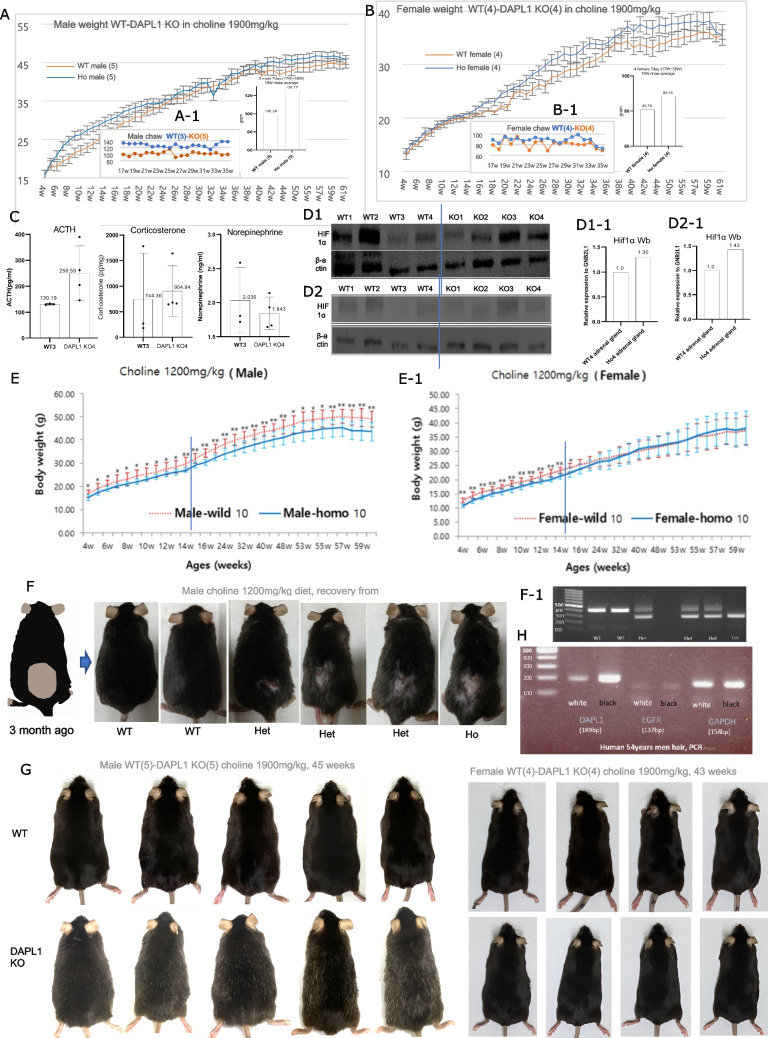
DAPL1 Knock-out mouse showed changes in body weight and hair according to changes in choline amount. (**A**) Average weight of 5 male mice that ate SNU chow (choline 1900 mg/kg, 4w-60w), (**A-1**) Average chow weight of 5 male mice that ate SNU chow (choline 1900 mg/kg), amount (bar graph) of chow eaten per week by 5 WT and 5 DAPL1 KO male mice between 17 and 35 weeks of age. (**B**) Average weight of 4 female mice that ate SNU chow (choline 1900 mg/kg, 4w-60w), (**B-1**) Average chow weight of 4 WT, 4 DAPL1 KO female mice that ate SNU chow (choline 1900 mg/kg), amount (bar graph) of chow eaten per week by 4 WT and 4 DAPL1 KO female mice between 17 and 35 weeks of age. **(C)** ACTH, Corticosterone, and Norepinephrine were measured in the plasma of male 3WT and 4DAPL1 KO mice using an ELISA kit. (**D1**, **D2**) Comparison of HIF1α protein expression in the adrenal gland of female 4WT and 4DAPL1 KO mice (Western blot). (**D1-1**, **D2-1**) Quantitative graph of D1,2 using Image J. (**E**) Comparison of body weights of male 10WT-10DAPL1 KO and (**E-1**) female 10WT-10DAPL1 KO mice raised in KRIBB (Choline 1200 mg/kg) from 4 to 60 weeks. (**F**) Six suffered nearly identical wounds to their backs from two large mice. Hair restoration after approximately 3 months in WT, hetero, and DAPL1 KO mice. (**F-1**) Genotyping results of six injured mice. (**G**) Comparison photos of hair in Wild type male 5, female 4, DAPL1 KO male 5, and female 4 fed SNU chow (choline 1900 mg/kg), an increase in white hair is confirmed in DAPL1 KO male 5. (**H**) Comparison of DAPL1 expression by PCR in white hair and adjacent black hair of a 54-year-old man

While raising DAPL1 KO mice, we noticed that the body weights of DAPL1 KO mice were slightly overweight (Fig. 4A and B). When measuring the amount of chow, it was discovered that KO mice ingested more chow (Fig. 4A-1 and B-1). It is known that when the stress hormone cortisol (corticosterone in rodents) increases, a mouse ingests more food [53] [54]. To determine whether the weight gain was due to corticosterone, ACTH, corticosterone, and norepinephrine were examined in mouse plasma using an ELISA kit. There was no difference in norepinephrine, but an increase in ACTH and corticosterone was confirmed (Fig. 4C). Based on these results, we speculated that the body weight increase in KO mouse might be due to elevated corticosterone and the related hypothalamic–pituitary–adrenal (HPA) axis. This prediction is in line with Chen et al.’s report regarding the relationship between the increase in testosterone and the hypothalamic-pituitary–gonadal (HPG) axis in DAPL1 KO mice [11].

If DAPL1 causes an increase of Fdft1, Pcyt1a, and Sptlc1, resulting in an increase in Cho, PC, and SM, which make up the outer membrane in the cell lipid bilayer. The increases of these lipids in the outer cell membrane lower oxygen permeability, possibly resulting in some hypoxia in the cells [55]. To confirm this hypothesis, the expression of HIF1α protein was examined in the adrenal glands (corticosterone production site) of mice. As expected, an increase of approximately 30% was observed in the KO compared to the WT (Fig. 4D1 and D1-1, D2 and D2-1)**.**

DAPL1 knockout (KO) mice were maintained at two independent facilities: the Korea Research Institute of Bioscience and Biotechnology (KRIBB) for phenotypic characterization and Seoul National University (SNU) for functional studies. Body weights were monitored from 4 to 60 weeks of age, revealing divergent outcomes between the two facilities (Fig. 4E and A, 4E-1 and B) (Supplement data: Table 4). Analysis of the respective mouse chows (Supplementary Table 5) indicated that differences in non-essential amino acids were unlikely to contribute to the phenotype, whereas the marked disparity in choline content (KRIBB: 1,200 mg/kg; SNU: 1,900 mg/kg) was the most plausible factor influencing body-weight variation. Choline is metabolized into betaine, acetylcholine, and multiple membrane phospholipid components, including phosphatidylcholine (PC), sphingomyelin (SM), lysophosphatidyl- choline (LPC), and Glycerophosphocholine (GPCho) [56]. Elevated dietary choline is associated with increased PC and SM levels. This observation is consistent with the effect of DAPL1, which upregulates *Fdft1*, *Pcyt1a*, and *Sptlc1*, resulting in increased cholesterol, PC, and SM. Elevated PC and cholesterol levels are known to markedly reduce membrane oxygen and water permeability [55, 57–59]. Thus, increased choline intake or DAPL1 activity is expected to promote intracellular hypoxia. To assess whether chow composition contributed to the observed weight differences, offspring (8 males and 8 females) from parents maintained on KRIBB chow (1,200 mg/kg choline) were allocated at 4 weeks of age into two groups (*n* = 4/sex each): one continued on KRIBB chow, while the other was switched to SNU chow (1,900 mg/kg choline). Body-weight divergence emerged at approximately 19 weeks in both sexes (Supplement Fig. 1A and B), suggesting that the interval between 4 and 19 weeks represents the period during which choline accumulation exerts its physiological effect. To verify whether the body-weight differences between KRIBB and SNU mice were attributable to dietary choline, a custom chow containing twice the choline level of the KRIBB diet was formulated. Because body weight can also be influenced by gut microbiota, new DAPL1 KO mice were generated from frozen sperm and reared at a third facility (Institute of Laboratory Animal Resources, Seoul National University). Consistent with previous observations, KO mice fed 1,200 mg/kg choline exhibited lower body weight than WT controls (Supplement Fig. 1C and D), whereas KO mice fed 2,400 mg/kg choline showed higher body weight compared with WT (Supplement Fig. 1C−1 and D-1), In the male group receiving 2,400 mg/kg choline, recurrent aggressive interactions were observed from approximately 14 weeks onward, resulting in two animals sustaining severe injuries that impacted their body-weight measurements (Supplementary Fig. 1C−1). Given a previous report of elevated testosterone in DAPL1 KO mice [11], we examined its potential contribution to body-weight phenotypes. Long-term testosterone exposure reduces body weight in males but increases it in females [60, 61]. As testosterone peaks at approximately 15 weeks in mice [62], comparisons beyond this age revealed opposing trends: body weight decreased in male DAPL1 KO mice and increased in female KO mice, as shown in Fig. 4A, E (males) and Fig. 4B, E-1 (females).

While breeding DAPL1 KO mice, we discovered abnormalities in hair. Eight male mice fed KRIBB chow (1200 mg choline/kg) were raised in two cages. The larger mouse in each cage chased the three smaller mice, biting them on the backs, causing extensive hair loss and wounds. Two mice were culled, and six mice with similarly sized wounds were raised. After about 3 months, the wound healed, but there was a large difference between individuals, so when genotyping was performed, it was confirmed that WT was fully recovered, Hetero was 1/2 recovered, and Homo was barely recovered (Fig. 4F, F-1). In male mice fed SNU chow (1900 mg choline/kg), an increase in gray hair was observed with age, and Fig. 4G shows this at 46 weeks of age. When the expression of DAPL1 was confirmed by PCR using white hair and surrounding black hair from a 54-year-old male, a significantly reduced level of DAPL1 was confirmed in the white hair (Fig. 4H). These evidences suggest that DAPL1 plays a significant role in hair regeneration and maintenance of black hair.

### Expected mechanisms of mouse weight difference and estimated DAPL1-LSM1 binding sequence

**Fig. 5:**
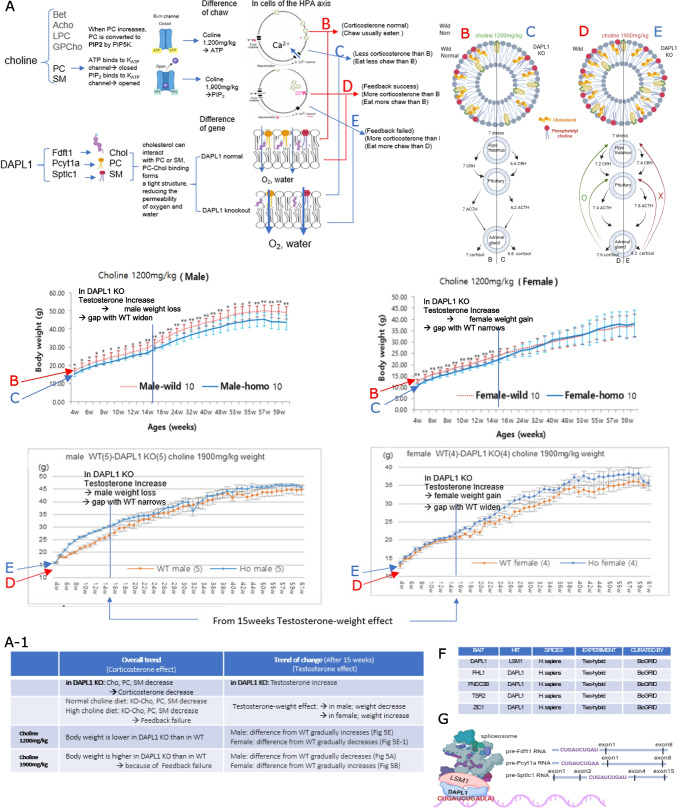
Expected diagram of mouse weight difference and estimated DAPL1-LSM1 binding sequence. (**A**) Schematic diagram explaining the cause of changes in body weight of DAPL1 KO mice according to changes in choline. Choline is used in the synthesis of Betaine (Bet), or acetylcholine (Acho), Lysophosphatidylcholine (LPC), Glycerophosphocholine (GPCho), Phosphatidylcholine (PC), and Sphingomyelin (SM). Among the metabolites of choline, PC and SM form the outer membrane in the cell double membrane, When PC increases above a certain amount in the cell membrane, it is converted to PIP_2_ by PIP5K, so when PC increases, PIP_2_ increases. K_ATP_ channels are distributed in the brain, heart, pancreas, kidneys, adrenal glands, and capillaries of the eyes and legs, etc. When ATP binds to the K_ATP_ channel, the channels close, and when PIP_2_ or PIP binds, the channel opens, releasing K^+^ out of the cell. (**A-1**) Table showing body weight changes in DAPL1 KO mice according to choline and testosterone content. (**B**, **C**) In the choline 1200 mg/kg diet, ATP binds to the K_ATP_ channels, so the channel is closed. There is a lot of K^+^ in the cell, and when an action potential occurs, a strong signal is generated, so many Ca channels are opened, and a lot of Ca^2+^ enters the cell, vesicle membrane ducking occurs well due to the action of Ca^2+^, so many hormones are released. (**D**, **E**) In the choline 1900 mg/kg diet, excess PC is converted to PIP_2_ by the action of PIP5K, and PIP_2_ increases in the cell membrane. PIP_2_ binds to the K_ATP_ channels, so the channel is opened. Because K^+^ flows out of the cell, and when an action potential occurs, a weak signal is generated, a small number of Ca channels are opened, and small numbers of Ca^2+^ enter the cell, because Ca.^2+^ is low, vesicle membrane ducking do not occur easily, so less hormones are released. The fact that DAPL1 increases the mRNA of Fdft1, Pcyt1a, and Sptlc1, ultimately leading to an increase in Chol, PC, and SM, is very similar to the function of choline. Cholesterol (Cho) can interact with PC and SM, and the combination of PC and Chol creates a very tight structure, as PC and Cho increase, oxygen and water permeation through the cell membrane decrease. In DAPL1 KO, less Chol, PC, and SM are produced than in WT, so oxygen and water permeability increases compared to WT, creating a lower stress environment inside the cell than in WT. (**B**) (choline 1200 mg/kg, Normal, Wild type, Standard) The cell membrane of the cells that make up the Hypothalamic–Pituitary–Adrenal Axis (HPA axis) contains a normal number of PC, SM, and Cholesterol. For example, when a stress stimulus of 7 comes from the outside, 7 CRH, 7 ACTH, and 7 Corticosterone are secreted. (**C**) (choline 1200 mg/kg, DAPL1 KO) The cell membrane of the cells that make up the HPA axis has less PC, SM, and Cholesterol than WT. Therefore, the permeability of oxygen and water into the cell increases, and the stress inside the cell decreases. So, the HPA axis reacts less than normal to external stimuli. Because mice produce less corticosterone, they eat less and have lower body weights than WT mice. (**D**) (choline 1900 mg/kg, Wild type) The cell membrane of the cells that make up the HPA axis has more PC and cholesterol than normal. So, at first, a lot of CRH, ACTH, and Corticosterone are produced, but through a feedback effect, they return to near normal. (**E**) (choline 1900 mg/kg, DAPL1 KO) The cell membrane of the cells that make up the HPA axis has less PC, SM, and Cholesterol than (**D**). Due to the failure of the feedback mechanism, large amounts of CRH, ACTH, and Corticosterone are produced. Because mice produce a lot of corticosterone, they eat a lot and have a higher body weight than WT. (**F**) The part related to DAPL1 in the contents of 52,414 human protein–protein interaction map (HuRI) reorganized in BioGRID. When DAPL1 is used as a beit, it shows that only LSM1 binds. (**G**) Putative DAPL1 binding sites of three RNAs (pre-Fdft1, pre-Pcyt1a, and pre-Sptlc1) predicted by PRIdictor [35]. When the three RNAs interact with protein LSM1, a similar binding site to the DAPL1-binding site was predicted in each of the RNAs. (The pictures above were created with BioRender)

We investigated the mechanisms underlying body weight changes in relation to dietary choline levels and DAPL1 expression. Variations in dietary choline appear to influence body weight differences in mice. On a 1,200 mg/kg choline diet, DAPL1 knockout (KO) mice exhibited low levels of Cho, PC, and SM. Under these conditions, the HPA axis receives sufficient oxygen and water, resulting in reduced stress hormone production, decreased food intake, and lower body weight compared to wild-type (WT) mice (Fig. 4E, E-1; Fig. 5B, C). In contrast, high-choline diets (1,900 or 2,400 mg/kg) elicited a modest stress hormone increase in WT mice via successful feedback regulation, whereas DAPL1 KO mice failed to regulate stress hormones, leading to body weight changes opposite to those observed on the 1,200 mg/kg diet (Fig. 4A, B; Fig. 5D, E; Supplementary Fig. 1C−1, D-1).

The mechanistic basis for this feedback failure involves the K_ATP_ channel, whose structure was elucidated in 2006 [63]. The channel closes upon binding of four ATP molecules to the C-terminal of the Kir domain and opens when PIP_2_ or PIP binds to the same region (Fig. 5A) [64](p155). Increased phosphatidylcholine (PC) in the membrane is hydrolyzed by phosphor lipase D (PLD) into phosphatidic acid (PA) and choline. PA activates phosphatidylinositol 4-phosphate 5-kinases (PIP5K), promoting PIP_2_ synthesis. Consequently, elevated membrane PC enhances PIP_2_ levels, modulating K_ATP_ channel activity [65].

On a high-choline diet (1,900 mg/kg), WT mice exhibit elevated PIP_2_ levels in the HPA axis cell membrane due to increased PC conversion (Fig. 5A). Upon stimulus-induced action potential, PIP_2_ binds to the K_ATP_ channel, opening it and promoting K^+^ efflux. This reduces membrane depolarization, limits Ca channel opening, decreases Ca^2+^ influx, and weakens hormone vesicle docking, ultimately reducing hormone release as part of normal feedback regulation (Fig. 5D).

In contrast, DAPL1 KO mice show reduced Pcyt1a expression, resulting in lower PC levels and consequently decreased PIP_2_ in the membrane (Fig. 5E). Upon stimulation, ATP binds the K_ATP_ channel, keeping it closed, which induces strong depolarization, opens numerous Ca channels, and increases Ca^2+^ influx. This enhances Ca^2+^-dependent vesicle docking and hormone release, indicating failure of feedback regulation (Fig. 5E).

When fed a low-choline diet (1,200 mg/kg), DAPL1 knockout (KO) mice exhibited lower body weight than WT mice (Fig. 4E, 4E-1). In contrast, on a high-choline diet (1,900 mg/kg), DAPL1 KO mice showed higher body weight than WT, demonstrating an opposite trend relative to the 1,200 mg/kg diet (Fig. 4A, B). These weight changes were both diet- and sex-dependent. On the 1,200 mg/kg diet, male DAPL1 KO mice progressively diverged from WT with age, whereas female DAPL1 KO mice gradually approached WT weight (Fig. 4E, 4E-1). On the 1,900 mg/kg diet, male DAPL1 KO mice gradually converged toward WT, whereas female DAPL1 KO mice diverged from WT (Fig. 4A, B). A summary of these trends is provided in Fig. 5A-1.

Previous studies reported elevated testosterone levels in DAPL1 KO mice [11]. Clinical evidence indicates that long-term testosterone administration reduces body weight in men [61], but increases weight in postmenopausal women [60], suggesting a sex-dependent effect. This aligns with the observed weight changes in DAPL1 KO mice. Specifically, on the 1,200 mg/kg diet, male KO mice showed gradual weight reduction, increasing the difference from WT (Fig. 5C male), while female KO mice gained weight, decreasing the difference from WT (Fig. 5C female). Conversely, on the 1,900 mg/kg diet, male KO mice decreased weight in periods of high testosterone, narrowing the gap with WT (Fig. 5E male), while female KO mice gained weight, widening the gap (Fig. 5E female). These observations indicate that DAPL1 influences body weight through choline-derived metabolites, PC and SM, and that testosterone contributes to the sex-dependent effects (Fig. 5B, C, D, E). Serum testosterone levels peak in reproductively active mice (~ 15 weeks) and decline with age (~ 65 weeks) [62].

We aimed to identify proteins that interact with DAPL1. We compared the function and expression of FHL2, FNDC3B, LSM1, TSR2, and ZIC1, proteins previously reported to interact with DAPL1 in databases such as HuRI, UniProt, BioGRID, and IntAct. Among these, U6 SnRNA-Associated Sm-Like Protein 1 (LSM1) was identified as the most likely DAPL1-binding protein. In the BioGRID dataset of the HuRI human binary protein interaction map [66], LSM1 was the only hit when DAPL1 was used as the bait (Fig. 5F). According to NCBI Gene, “LSM proteins form stable heteromers that bind specifically to the 3'-terminal oligo(U) tract of U6 snRNA and may facilitate pre-mRNA splicing by mediating U4/U6 snRNP formation” [67].

Based on these reports, we hypothesized that DAPL1 interaction with LSM1 could enhance spliceosome activity by binding to pre-mRNAs of Fdft1, Pcyt1a, and Sptlc1. We used PRIdictor, a protein-RNA binding prediction tool, to identify the RNA-binding sites of DAPL1 and LSM1 [35], we identified potential DAPL1-LSM1 binding sites in pre-Fdft1, pre-Pcyt1a, and pre-Sptlc1. Despite notable differences in protein sequences between DAPL1 and LSM1, the predicted pre-RNA binding sites overlapped, in 3 pre-mRNA, the motif CUGAUCUGAU(A) arrangement showed a high score in common for binding to DAPL1 and LSM1 proteins (Fig. 5G).

## Discussion

DAPL1 is mainly expressed in keratinized tissues such as the skin, nails, hair follicles, tongue, esophagus, vagina and cornea [4] (GTExPortal). In non-keratinized tissues, expression has been reported in melanocytes of the skin and hair that synthesize melanin, retinal pigment epithelium (RPE), and cells that produce catecholamines (dopamine, epinephrine, norepinephrine), such as kidney, adrenal glands, substantia nigra, hypothalamus [23, 24], and testicular Leydig cells. It is highly expressed in eye [11]**.** Keratinization, melanin, catecholamine synthesis enzymes, and the eye all have one thing in common: they require a slightly acidic state.

In this study, we demonstrated that Np63 and GRα monomeric transcription factors can regulate DAPL1. DAPL1, in turn, regulates Fdft1, Pcyt1a, and Sptlc1, leading to an increase in Cho, PC, and SM, which in turn decrease the oxygen permeability of the cell membrane and make the cytoplasm slightly acidic.

Our theory is supported by the finding that DAPL1 overexpression causes macular degeneration (AMD) [5]. and that macular drusen from 7 AMD patients contain 37.47% esterified cholesterol, 36.89% phosphatidylcholine, and 24.24% sphingomyelin [68]**.** DAPL1 is highly expressed in the eye, an organ that relies primarily on cytoplasmic glycolysis for energy due to the absence of most organelles—and even nuclei in the lens—to maximize light transmittance. Given this high DAPL1 expression, elevated levels of cholesterol, PC, and SM would be expected relative to other tissues, thereby reducing membrane oxygen permeability and protecting the eye from excessive oxygen exposure [69]. In Jurkat T cells lacking endogenous GRα, expression of DAPL1 was increased by GRα, GRαA, GRαB, and GRαC, but not by GRαD [70]. GRα contains a ligand-binding domain, DNA-binding domain, and N-terminal activation domain, and typically functions as a dimer; mutations in the ligand- or DNA-binding domains prevent dimerization, producing monomers. In osteosarcoma cells transfected with domain-specific GRα mutants and treated with dexamethasone, the DAPL1 locus showed 8–9 transcriptional leads with wild-type or activation-domain–mutant GRα (dimeric forms), but 17 and 22 leads with ligand-binding– or DNA-binding–domain mutants (monomeric forms) [71]. These findings collectively indicate that DAPL1 expression is preferentially driven by monomeric, rather than dimeric, GRα.

Collectively, DAPL1 promotes cancer cell proliferation and helps maintain body weight under a normal choline diet. Furthermore, consistent with reported increases in testosterone in DAPL1 KO mice [11], our longitudinal analysis confirmed that testosterone reduces body weight in males while increasing it in females [60, 61]. The results presented in the above papers demonstrate that the content and inferences of this paper are accurate.

## Supplementary information

Below is the link to the electronic supplementary material.

ESM 1 (DOCX 132 KB)

## Data Availability

The TCGA datasets used in this study are accessible from FIREHOSE (Broad GDAC Firehose) and the datasets generated during this study are available from the corresponding author upon reasonable request.
