# Supplementary material for: DAPL1 is activated by Np63 and GRα and regulates lipid metabolism
Source: J Mol Med (Berl). 2026 Jan 31;104(1):37. doi: 10.1007/s00109-025-02636-8 (PMC12860865; doi:10.1007/s00109-025-02636-8)
Supplement: Supplementary file 1 — (DOCX 132 KB) [file 109_2025_2636_MOESM1_ESM.docx]

**Supplement data**

**Table 1.** Mass parameters for lipidomics.

| **Agilent 6530 hybrid quadrupole Time of Flight Mass Spectrometer** | |
| --- | --- |
| **Mass parameters** | |
| **MS1** |  |
| ESI polarity | Positive, negative |
| MS resolution | 4 Ghz |
| Capillary voltage | 3.5 kV |
| MS1 range | 300~1200 m/z |
| Nozzle voltage | 1000 V |
| Fragmentor voltage | 135 V |
| Gas temperature | 325℃ |
| Drying gas | 8 L/min |
| Nebulizer gas | 35 psi |
| Sheath gas temperature | 350℃ |
| Sheath gas flow | 11 L/min |
| MS1 acquisition speed | 5 spectra/s |
| **MS2 (AutoMS/MS)** |  |
| MS1 range | 50~1200 m/z |
| Collision energy | 20 eV |
| Isolation window | Narrow (1.3 m/z) |
| Precursor ions per cycle | 4 |
| MS2 acquisition speed | 8 spectra/s |
|  |  |

**Table 2.** LC-MS/MS data-processing parameters in MSDIAL.

| **MS-DIAL** | |
| --- | --- |
| Data collection | |
| MS1 tolerance | 0.03 Da |
| MS2 tolerance | 0.05 Da |
| Retention time begin | 0 min |
| Retention time end | 16 min |
| Mass range begin | 300 Da |
| Mass range end | 1200 Da |
| Maximum charged number | 2 |
| Peak detection | |
| Minimum peak height | 1000 amplitude |
| Mass slice width | 0.1 Da |
| Smoothing method | Linear weighted moving average |
| Smoothing level | 3 scan |
| Minimum peak width | 5 scan |
| MS2 Dec | |
| Sigma window value | 0.1 |
| MS/MS abundance cut off | 0 amplitude |
| Exclude after precursor ion | checked |
| Keep the isotopic ions until | 0.5 Da |
| Identification | |
| Retention time tolerance | 100 min |
| Accurate mass tolerance (MS1) | 0.03 Da |
| Accurate mass tolerance (MS2) | 0.05 Da |
| Identification score cut off | 80% |
| Adduct | |
| Positive: [M+H]+, [M+NH4]+, [M+Na]+, [M+H-H2O]+, [ [2M+H]+ | |
| Negative: [M-H]-, [M-H2O-H]-, [M+Cl]-, [2M-H]- | |
| Alignment | |
| Reference file | one of QC sample file |
| Retention time tolerance | 0.05 min |
| MS1 tolerance | 0.015 Da |
| Blank filter: sample max / blank average | 3 fold change |

**Table 3.10 Genes that move like DAPL1**

**Table 4. DAPL1 KO mouse weight change table provided by KRIBB**

1. growth curve (4w-60w)

As a result of continuously measuring the body weight of Dapl1 homo mutant mice from 4 to 60 weeks of age, male mice were lower than the control group over the entire measurement period, female mice were temporarily measured to have lower body weights than controls from 4 to 15 weeks of age (Result Fig. 4E, E-1, Result Table1)

As a result, 1-year-old Dapl1 homo mutant mice had a consistently lower body weight than the control group, it is judged that the female mice had a lower body weight than the control group only during the period of 15 weeks of age or less, and then recovered.

**Result Table 1-1. Average body weight change of Dapl1 KO mice**

| **sex** | **genotype** | | **weight by age (g)** | | | | | | |
| --- | --- | --- | --- | --- | --- | --- | --- | --- | --- |
|  |  |  | **4w** | **5w** | **6w** | **7w** | **8w** | **9w** | **10w** |
| Male | Wild | average | 16.99 | 19.28 | 20.81 | 22.32 | 23.33 | 24.49 | 25.38 |
|  |  | ± SD | 1.61 | 2.07 | 1.94 | 2.73 | 2.71 | 2.57 | 2.65 |
|  | Homo | average | 15.37* | 17.46* | 19.06* | 20.29* | 21.20* | 22.09* | 23.25* |
|  |  | ± SD | 1.28 | 1.25 | 1.09 | 1.06 | 1.00 | 1.15 | 1.02 |
| Female | Wild | average | 12.63 | 14.46 | 15.65 | 16.49 | 17.44 | 18.57 | 19.39 |
|  |  | ± SD | 0.89 | 1.16 | 1.10 | 0.96 | 0.90 | 1.01 | 1.21 |
|  | Homo | average | 10.87** | 12.61** | 13.80** | 14.98** | 15.77** | 16.64** | 17.59** |
|  |  | ± SD | 0.60 | 0.90 | 0.72 | 1.11 | 1.12 | 1.16 | 1.18 |

*, P<0.05; **, P<0.01. n=10 (male), 10(female)

**Result Table 1-2. Average body weight change of Dapl1 KO mice**

| **sex** | **genotype** | | **weight by age (g)** | | | | | |
| --- | --- | --- | --- | --- | --- | --- | --- | --- |
|  |  |  | **11w** | **12w** | **13w** | **14w** | **15w** | **16w** |
| Male | Wild | average | 26.47 | 27.54 | 28.45 | 29.65 | 32.03 | 34.25 |
|  |  | ± SD | 2.70 | 2.56 | 2.57 | 2.66 | 2.07 | 1.82 |
|  | Homo | average | 24.25* | 25.47* | 26.29* | 26.98** | 29.16** | 30.31** |
|  |  | ± SD | 1.19 | 1.14 | 1.14 | 0.85 | 1.39 | 1.37 |
| Female | Wild | average | 20.12 | 21.19 | 22.18 | 23.21 | 24.09 | 25.21 |
|  |  | ± SD | 0.95 | 1.27 | 1.27 | 1.05 | 1.43 | 1.83 |
|  | Homo | average | 18.57** | 19.41** | 20.14** | 21.16** | 22.53* | 23.67 |
|  |  | ± SD | 0.94 | 0.96 | 0.95 | 1.08 | 1.60 | 1.66 |

*, P<0.05; **, P<0.01. n=10 (male), 10(female)

**Result Table 1-3. Average body weight change of Dapl1 KO mice**

| **sex** | **genotype** | | **weight by age (g)** | | | | | |
| --- | --- | --- | --- | --- | --- | --- | --- | --- |
|  |  |  | **20w** | **24w** | **28w** | **32w** | **36w** | **40w** |
| Male | Wild | average | 36.26 | 38.39 | 40.03 | 41.00 | 42.31 | 43.62 |
|  |  | ± SD | 1.85 | 1.97 | 2.44 | 2.40 | 2.18 | 2.17 |
|  | Homo | average | 32.21** | 34.13** | 35.46** | 36.89** | 38.09** | 39.23** |
|  |  | ± SD | 1.27 | 2.43 | 2.37 | 2.60 | 2.70 | 2.89 |
| Female | Wild | average | 26.06 | 27.06 | 27.62 | 28.49 | 29.33 | 30.12 |
|  |  | ± SD | 1.99 | 2.54 | 2.63 | 2.98 | 3.11 | 3.27 |
|  | Homo | average | 24.96 | 26.18 | 26.79 | 27.92 | 29.17 | 30.70 |
|  |  | ± SD | 1.89 | 2.70 | 3.32 | 3.16 | 3.68 | 3.47 |

**, P<0.01. n=10 (male), 10 (female)

**Result Table 1-4. Average body weight change of Dapl1 KO mice**

| **sex** | **genotype** | | **weight of age (g)** | | | | | |
| --- | --- | --- | --- | --- | --- | --- | --- | --- |
|  |  |  | **44w** | **48w** | **52w** | **53w** | **54w** | **55w** |
| Male | Wild | average | 44.29 | 45.68 | 46.76 | 48.14 | 48.67 | 49.25 |
|  |  | ± SD | 2.75 | 3.08 | 3.74 | 3.90 | 3.56 | 3.59 |
|  | Homo | average | 40.27** | 41.25** | 42.46* | 43.43* | 44.07* | 44.78* |
|  |  | ± SD | 3.14 | 3.49 | 3.44 | 3.79 | 3.74 | 3.98 |
| Female | Wild | average | 30.72 | 31.41 | 32.17 | 32.84 | 33.96 | 35.34 |
|  |  | ± SD | 3.32 | 3.64 | 3.93 | 3.98 | 4.13 | 4.30 |
|  | Homo | average | 31.40 | 31.91 | 32.69 | 33.17 | 34.10 | 35.52 |
|  |  | ± SD | 3.61 | 3.52 | 3.75 | 4.41 | 5.65 | 5.80 |

*, P<0.05; **, P<0.01. n=10 (male), 10(female)

**Result Table 1-5. Average body weight change of Dapl1 KO mice**

| **sex** | **genotype** | | **weight of age (g)** | | | | |
| --- | --- | --- | --- | --- | --- | --- | --- |
|  |  |  | **56w** | **57w** | **58w** | **59w** | **60w** |
| Male | Wild | average | 49.72 | 50.22 | 49.60 | 49.68 | 48.95 |
|  |  | ± SD | 2.92 | 2.93 | 3.20 | 3.76 | 3.47 |
|  | Homo | average | 45.01** | 45.35** | 44.09** | 44.02** | 43.56** |
|  |  | ± SD | 3.75 | 3.91 | 3.85 | 4.31 | 3.88 |
| Female | Wild | average | 35.27 | 35.95 | 37.01 | 36.75 | 37.29 |
|  |  | ± SD | 4.90 | 5.18 | 4.58 | 4.83 | 4.96 |
|  | Homo | average | 36.51 | 37.45 | 37.79 | 37.34 | 38.03 |
|  |  | ± SD | 5.71 | 5.67 | 5.62 | 6.02 | 6.06 |

**, P<0.01. n=10 (male), 10(female)

**Table 5. Comparison table of Elements of mouse chaw used by KRIBB and SNU**.

|  | **Elements** | **KRIBB** | **SNU** |  |  | **Elements** | **KRIBB** | **SNU** |
| --- | --- | --- | --- | --- | --- | --- | --- | --- |
| Macronutrients | Protein | 18.60% | 18% |  | Minerals | Calcium | 1.00% | 1.00% |
|  | Fat | 6.20% | 5.00% |  |  | Phosphorus | 0.70% | 0.85% |
|  | Fiber | 3.50% | 5.00% |  |  | Potassium | 0.60% | 0.55% |
|  | Ash | 5.30% | 8.00% |  |  | Sodium | 0.20% | 0.25% |
|  |  |  |  |  |  | Chloride | 0.40% |  |
| Amino Acid | Arginine | 1.00% | 0.90% |  |  | Magnesium | 0.20% | 0.15% |
|  | Alanine | 1.10% |  |  |  | Iron | 200mg/kg | 300ppm |
|  | Aspartic Acid | 1.40% |  |  |  | Zinc | 70mg/kg | 40ppm |
|  | Cystine | 0.30% | 0.25% |  |  | Manganese | 100mg/kg | 140ppm |
|  | Glycine | 0.80% | 0.95% |  |  | Copper | 15mg/kg | 12ppm |
|  | Glutamic Acid | 3.40% |  |  |  | Cobalt |  | 0.7ppm |
|  | Histidine | 0.40% | 0.38% |  |  | Selenium | 0.23mg/kg |  |
|  | Isoleucine | 0.80% | 0.95% |  |  | Iodine | 6mg/kg | 1.8ppm |
|  | Leucine | 1.80% | 1.40% |  |  |  |  |  |
|  | Lysine | 1.10% | 0.85% |  | Vitamins | Vitamin A | 30IU/g | 17.0IU/g |
|  | Methionine | 0.60% | 0.35% |  |  | B1 (thiamin) | 117mg/kg | 15ppm |
|  | Phenylalanine | 1.00% | 0.85% |  |  | B2 (Riboflavin) | 27mg/kg | 9ppm |
|  | Proline | 1.60% |  |  |  | B6 (pyridoxine) | 26mg/kg | 10ppm |
|  | Serine | 1.10% |  |  |  | B12 (cyanocobalamin) | 0.15mg/kg | 75Mcg/kg |
|  | Threonine | 0.70% | 0.65% |  |  | Niacin | 115mg/kg | 70ppm |
|  | Tryptophan | 0.20% | 0.20% |  |  | D3 | 2.0IU/g | 4.0IU/g |
|  | Tyrosine | 0.60% | 0.60% |  |  | E (alpha-tocopherol) | 135IU/kg | 45IU/kg |
|  | Valine | 0.90% | 0.90% |  |  | K |  | 2.0ppm |
|  |  |  |  |  |  | K3 (menadione) | 100mg/kg |  |
|  |  |  |  |  |  | Pantothenic Acid | 140mg/kg | 30ppm |
|  |  |  |  |  |  | Biotin | 0.90mg/kg | 0.20ppm |
|  |  |  |  |  |  | Folate | 9mg/kg | 2.0ppm |
|  |  |  |  |  |  | Choline | 1200mg/kg | 1900ppm |

### Table 6. Primers used for the RT-PCR. PCR

**Supplement Figure 1 The body weight of DAPL1 KO mice varies depending on the amount of Choline.**

A

B

KRIBB

chaw

Baby birth

Father, Mother

4weeks

KRIBB

chaw

SNU

chaw

35weeks


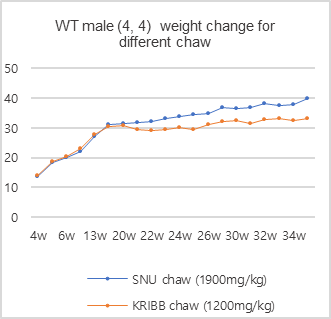


C-1

C

D

D-1
